# Exploring the vaccine conversation on TikTok in Italy: beyond classic vaccine stances

**DOI:** 10.1186/s12889-023-15748-y

**Published:** 2023-05-12

**Authors:** Lorenza Parisi, Simone Mulargia, Francesca Comunello, Vittoria Bernardini, Arianna Bussoletti, Carla Rita Nisi, Luisa Russo, Ilaria Campagna, Barbara Lanfranchi, Ileana Croci, Eleonora Grassucci, Francesco Gesualdo

**Affiliations:** 1 Human Sciences Department, Link Campus University, Rome, Italy; 2 Department of Human Studies - Communication, Education, and Psychology, LUMSA University, Borgo S. Angelo, 13, Rome, 00193 Italy; 3 Department of Communication and Social Research, Sapienza University of Rome, Piazzale Aldo Moro 5, Rome, 00185 Italy; 4 Predictive and Preventive Medicine Research Unit, Bambino Gesù Children’s Hospital IRCCS, Piazza S. Onofrio 4, Rome, 00165 Italy; 5 Department of Information Engineering, Electronics, and Telecommunication, Sapienza University of Rome, Piazzale Aldo Moro 5, Rome, 00185 Italy

**Keywords:** Vaccines, COVID-19, Vaccine hesitancy, Communication, Social media, TikTok, Irony

## Abstract

TikTok, a social media platform for creating and sharing short videos, has seen a surge in popularity during the COVID-19 pandemic. To analyse the Italian vaccine conversation on TikTok, we downloaded a sample of videos with a high play count (Top Videos), identified through an unofficial Application Programming Interface (consistent with TikTok’s Terms of Service), and collected public videos from vaccine sceptic users through snowball sampling (Vaccine Sceptics’ videos). The videos were analysed using qualitative and quantitative methods, in terms of vaccine stance, tone of voice, topic, conformity with TikTok style, and other characteristics. The final datasets consisted of 754 Top Videos (by 510 single users) plus 180 Vaccine Sceptics’ videos (by 29 single users), posted between January 2020 and March 2021. In 40.5% of the Top Videos the stance was promotional, 33.9% were indefinite-ironic, 11.3% were neutral, 9.7% were discouraging, and 3.1% were ambiguous (i.e. expressing an ambivalent stance towards vaccines); 43% of promotional videos were from healthcare professionals. More than 95% of the Vaccine Sceptic videos were discouraging. Multiple correspondence analysis showed that, compared to other stances, promotional videos were more frequently created by healthcare professionals and by females, and their most frequent topic was herd immunity. Discouraging videos were associated with a polemical tone of voice and their topics were conspiracy and freedom of choice. Our analysis shows that Italian vaccine-sceptic users on TikTok are limited in number and vocality, and the large proportion of videos with an indefinite-ironic stance might imply that the incidence of affective polarisation could be lower on TikTok, compared to other social media, in the Italian context. Safety is the most frequent concern of users, and we recorded an interesting presence of healthcare professionals among the creators. TikTok should be considered as a medium for vaccine communication and for vaccine promotion campaigns.

**Supplementary Information:**

The online version contains supplementary material available at 10.1186/s12889-023-15748-y.

## Introduction

Social media have recently drawn the attention of the scientific community as an important source of information on vaccine acceptance and as a potential means for improving health literacy on vaccines, planning communication interventions targeting different populations, and promoting healthy behaviours and vaccine uptake [1–3]. Social media have also been studied as the main avenue of circulation for disinformation and misinformation, including conspiracy theories [4], which may trigger vaccine hesitancy and increase the risk of vaccine preventable diseases epidemics [5–7]. The overabundance of information - including misleading information - has worsened during the COVID-19 pandemic, and has been defined as an infodemic [8, 9]. An infodemic can impair the ability of web and social media users to find trustworthy sources of information [10, 11], and may impact on people’s vaccination choices [12].

During the COVID-19 pandemic, social media users have increased and so has the time spent online. Italian data show that social media users have increased by 6.7% in 2021 from 2020, growing to 76.6% of the general population [13]. The increase was recorded on existing platforms, like Facebook, and on newer platforms, like TikTok.

TikTok is a social media platform for creating and sharing short videos. Launched in China in 2016 and made available globally in 2017, it has rapidly surged in popularity, especially among young users. TikTok’s popularity steeply rose during the COVID-19 pandemic, as the app crossed the two-billion-download mark [14]. In February 2021, TikTok counted 100 million active users [15], who increased to 1 billion in September 2021 [16]. As of 2021, the 18–24 age group was the most represented among TikTok users (34.9%) [17]. In Italy, 34,5% of young people (14–29) use TikTok [13].

TikTok’s algorithms (i.e. the mechanisms determining what content will appear on the user’s feed) are considered very effective in suggesting personalised videos and, therefore, in generating users’ engagement on very specific topics; however, the company has not revealed any information about their mechanisms. Users have turned to social media platforms, including TikTok, to gather and spread information about the pandemic, including unreliable information and fake news, which brought the platform to collaborate with the World Health Organization (WHO) to implement anti-fake news strategies [18].

Recently, scholars have started analysing the circulation of health-related content on TikTok, mainly focusing on videos produced by official health accounts [19] and healthcare workers [20]. Studies have explored the emerging use of TikTok during the COVID-19 pandemic from a public health perspective, investigating the topics addressed by the most popular videos [21]. Studies have also addressed the factors driving citizen engagement to improve health information dissemination [22], and the best ways to use social media during pandemics [23]. A structured analysis of the vaccine conversation on TikTok is needed to better understand this ecosystem from the public health perspective, to elucidate TikTok users’ perception and understanding of vaccines, and to explore tones of voice and narratives used in videos with vaccine-related content. Such an analysis can also contribute to better understanding the role of playful and humorous videos addressing scientific topics and shed light on this platform’s potential as a channel for better exploring young people’s expectations and fears toward vaccines. To this aim, we investigated the way TikTok has been used to talk about vaccines in Italy up to March 2021, by analysing vaccine-related videos in terms of vaccine stance, actors involved in the conversation, content, prevalent language and tone of voice, most used hashtags, and conformity to the platform’s style.

## Materials and methods

This is a cross-sectional, retrospective study analysing the vaccine discourse on TikTok in Italy through the analysis of videos published on the platform between January 2020 and March 2021. The Italian vaccine programme started rolling out in late December 2020.

### Exploratory phase

First, we conducted an exploratory analysis of vaccine-related videos on Tiktok. Each researcher created a new account and, without setting any preferences (to avoid algorithmic personalisation), we screened videos in the “hashtags” and “top videos” sections, identified through the TikTok search engine using the keyword “vaccino” (“vaccine” in Italian). Furthermore, we explored the comments on the videos and recorded vaccine-related hashtags used in the videos’ descriptions.

From this initial screening, we acquired the following information:

- most of the videos tagged with vaccine-related hashtags had pro-vaccine content;
- most vaccine-hesitant users expressed their ambiguous/ambivalent vaccine stance in comments on other users’ videos rather than on videos made by themselves;
- most videos with clear vaccine sceptic content used generic hashtags rather than hashtags clearly expressing a vaccine-discouraging stance; only a minority of the videos with a discouraging stance towards vaccines used the hashtag #novax.

This last finding is probably due to the fact that, as of December 2020, anti-vax content published on TikTok risks being banned, either by the platform or upon reporting by other users. Therefore, some users try to make their content less visible in order to protect it, e.g. by marking the videos with hashtags that could not be easily associated with vaccines (such as #opinion, #democracy, #freedom, #liar etc.). Some creators simply used a custom hashtag identifying the single content creators, allowing for the videos to be found by specific users without explicitly describing their content.

Based on these preliminary findings, we developed the structured method described in the following sections.

### Data retrieval

Data was downloaded through an Unofficial TikTok Application Programming Interface (API) Wrapper in Python [24], after ensuring that the process was compatible with TikTok’s Terms of Service. We used the Python API feature, which enables the user to download information on TikTok videos and users, searching by hashtag. The following data were available from the API: video URL, textual description of the video, timestamp, user’s description, challenge title (a challenge is a trend in which creators are invited to perform specific actions in the video), share count, comment count, play count and hashtags.

We retrieved relevant videos from the API using a search filter that included five different vaccine-related hashtags in Italian. Relevant hashtags were identified during the exploratory phase and additional hashtags were incorporated based on initial search results from the API.

The final list of hashtags included the following: #vaccini (600), #vaccino (4000), #vaccinocovid (300), #vaccinocovid19 (700), #vaccinoanticovid (3500), #novax (700).

Using these hashtags, we selected the 1000 videos with the highest play count value (i.e. “Top Videos”). Only publicly available videos were downloaded and stored.

### Manual selection of vaccine sceptics’ videos

Based on the exploratory findings, we decided to further explore videos expressing a discouraging stance to better understand the vaccine sceptic community. We used a snowball sampling technique [25, 26] to manually identify a pool of vaccine sceptic users. We first viewed the videos obtained through the API and their comments to identify users with a discouraging stance towards vaccines. We then explored their profile to find more vaccine sceptic users among their contacts. We also conducted additional searches using hashtags not directly related to vaccines but commonly used by creators with a discouraging stance. We collected public vaccine-related videos posted by this pool of users, and downloaded them on a daily basis between January and March 2021. Only publicly available videos were downloaded and stored. Hereafter, we will refer to this set of videos as “Vaccine Sceptics’ videos”.

### Coding

We analysed the videos through video-based content analysis [27]. We created a coding book that included variables generated both deductively and inductively [28]. For the final list of variables considered in the video-based content analysis, see Table 1. Information on the type of user was obtained from the profile’s bio. Vaccine stance categories are discussed in more depth in the next section.

Since our research team is composed of researchers from the field of healthcare (FG, BL, LR, IC) and from social sciences (LP, FC, VB, CRN, AB), each video was coded independently by two researchers from each field. This helped ensure reliability of the analysis by including different disciplinary perspectives. The pairs of researchers who classified the same video subsequently reviewed discrepancies in the classification for each variable jointly and, through discussion, agreed on a common classification, reaching inter-coder agreement. Additionally, during the classification process, the research team conducted weekly meetings to cyclically refine common classification rules, collegially review ambiguous videos and discuss ongoing findings. This ensured that all researchers were in full agreement on the coding categories. Furthermore, as researchers from different disciplines, we were able to collectively share our interpretations of the data from different perspectives, allowing for a multi-faceted understanding of the material.

If the video was off-topic/private/had been cancelled or made private between the day of the download and the day on which it was classified, the video was excluded from the analysis.

**Table 1:** Information recorded for each video

Category	Variables	Values
Type of user	Gender	male, female, other
	User’s profession	healthcare, media and journalism, other
	Number of followers	discrete variable
Video features	Play count	discrete variable
	Share count	discrete variable
	Comment count	discrete variable
	Presence of COVID-19 banner or COVID-19 vaccine banner generated by TikTok and directing to the WHO website	yes/no
	Q&A style (displaying a comment to which the creator responds to)	yes/no
	Trend or challenge style (a typical kind of TikTok video with a specific format in terms of music, movements and/or dance, typically inviting people to replicate it)	yes/no
	Engaging music - lip sync	yes/no
	Video format	Face to Camera videos, videos or images with on-screen text, videos without text, images without text, infographic
Vaccine-related	Type of vaccine	anti-COVID-19, other
	Information Source	non scientific institutions, scientific institutions, mainstream media, social media post, no source reported, source reported but unspecified
	Topic	safety, efficacy, herd immunity, strategy, conspiracy, freedom of choice, health literacy, other (if more than one topic was included in the video, the researcher indicated the prevalent one)
	Personal storytelling about vaccine (e.g. “today I received the vaccine”)	yes/no
	Tone of voice	ironic, questioning, polemical/complaining, worried, supportive/empathic, encouraging, enthusiastic, neutral, other (if the video could be described with more than one tone of voice, the researcher indicated the prevalent one)
	Stance	promotional, neutral, discouraging, ambiguous, indefinite/ironic

### Vaccine stance definitions

In order to describe the vaccine stance of each video, we initially adapted the vaccine stance categorisation used by Martin et al. [29]: promotional, discouraging, ambiguous, and neutral. During the exploratory phase, we realised that these categories, originally used for tweets, were not completely adaptable to the content we found on Tiktok. Therefore, we created a new category, “indefinite-ironic”, that was attributed to videos that had no clearly identifiable stance, and that referred to vaccines in an ironic way (we further discuss this choice in the Results and Discussion sections). The final list of vaccine stance categories are defined as in Table 2.

**Table 2:** Stance categories and definitions

**Promotional** Videos communicate public health benefits or safety of vaccination. Videos encourage vaccination. Describes risks of not vaccinating Posts refute claims that vaccines are dangerous.
**Ambiguous** Content contains indecision, uncertainty on the risks or benefits of vaccination. Contains disapproving and approving information.
**Neutral** Contains no elements of uncertainty, promotional or negative content. These are often statements. This includes factual recommendations about vaccines.
**Discouraging** Contains negative attitudes/arguments against vaccines. Contains questions re. effectiveness/safety or possibility of adverse reactions that may or may not be proven. Discourages the use of recommended vaccines.
**Indefinite-Ironic** Has no clear identifiable stance: does not clearly promote or discourage vaccines, does not have elements of uncertainty, nor does it have a clearly neutral stance. Has an ironic, humorous nature. Typically refers to a challenge or a trend.

### Statistical analysis

Categorical variables were tabulated as frequencies and valid percentages, while discrete variables were presented using median and interquartile range (IQR), since the variables were non-normally distributed. Normality was assessed both graphically and using the Shapiro-Wilks test. Multiple correspondence analysis was used to identify variables associated with stance. The following variables were used to generate the graph (“active” variables): tone of voice, stance, user’s gender, topic, and user’s profession.

Data analysis was carried out using STATA 17.0 MP (StataCorp, College Station, Texas, USA).

## Results

From the API, we collected a total of 8582 vaccine-related videos, published between January 2020 and March 2021 in Italian. From this dataset, we selected the top 1000 videos in terms of play count; 156 were excluded as they were off-topic, 63 had been deleted, 10 had been made private at the time the video URL was accessed, and, for 17 videos, the user had been deleted. The Top Video final dataset included 754 videos, posted by 510 unique users.

Through snowball sampling, we collected a total of 193 Vaccine Sceptics’ videos. Of these, five were not included as they were off-topic, and eight were not available at the time of the analysis. The final Vaccine Sceptics’ videos dataset included 180 videos, by 29 single users. Table 3 shows single users’ characteristics by dataset. They were prevalently male (54.9%), females accounted for 36.8%, while in 43 cases the user’s gender could not be defined (e.g. an organisation, or a couple/family). For 83.3% of users, their profession was not stated nor understandable from their profile; 13.4% were healthcare professionals and 3.3% were media professionals.

**Table 3:** Users’ characteristics by dataset

	Top videos (n = 510)		Vaccine sceptics (n = 29)		Total (n = 539)	
	n	%	n	%	n	%
**Sex**						
Male	268	54.6%	16	61.5%	284	54.9%
Female	186	37.9%	4	15.4%	190	36.8%
Other (profiles from organisations, families etc.)	37	7.5%	6	23.1%	43	8.3%
**User’s profession**						
Healthcare professional	71	13.9%	1	3.6%	72	13.4%
Media and journalism	18	3.5%	0	0.0%	18	3.3%
Other	421	82.5%	27	96.4%	448	83.3%
**User’s followers, median (IQR)**	16153.5 (2947–59,300)		1022 (162–3503)		14,600 (2581–48,300)	

Table 4 shows video characteristics by dataset.

The Top Videos were created between 20 January 2020 and 16 March 2021. The Vaccine Sceptics’ videos were created between 5 September 2020 and 20 March 2021.

**Table 4:** Video characteristics by dataset

	Top Videos (n = 754)		Vaccine Sceptics (n = 180)		Total (n = 934)	
	n	%	n	%	n	%
**Number of single users**	510		29		539	
**Play count, median (IQR)**	24,600 (12,800–56,200)		320 (10–2018)		19,600 (10,800–50,300)	
**Share count, median (IQR)**	58 (17–224)		2 (0–16)		51 (12–195)	
**Comment count, median (IQR)**	73 (28–180)		2 (0–8)		57 (15–163)	
**COVID-19 or COVID-19 vaccine banner**	579	76.8%	47	26.1%	626	67.0%
**Q&A style**	108	14.3%	19	10.6%	127	13.6%
**Trend or challenge style**	157	20.8%	0	0.0%	157	16.8%
**Engaging music/lip sync**	397	52.6%	79	43.9%	476	51.0%
**Video format**						
Face to Camera	512	67.9%	28	15.6%	540	57.8%
Video or image with on-screen text	208	27.6%	114	63.3%	322	34.5%
Video without text	22	2.9%	10	5.6%	32	3.4%
Image without text	5	0.7%	12	6.6%	17	1.8%
Infographic	7	0.9%	16	8.9%	23	2.5%
**Type of vaccine**						
anti-COVID-19	694	92.0%	171	95.0%	865	92.6%
other	60	8.0%	9	5.0%	69	7.4%
**Information source**						
Social media posts	13	1.7%	17	9.4%	30	3.2%
Mainstream media	73	9.7%	90	50.0%	163	17.5%
Institutions (non scientific)	9	1.2%	3	1.7%	12	1.3%
Institutions (scientific)	17	2.3%	3	1.7%	20	2.1%
Unspecified source of information	26	3.5%	14	7.8%	40	4.3%
No information source reported	615	81.6%	53	29.4%	668	71.5%
**Video’s topic**						
Safety	378	50.1%	93	51.7%	471	50.4%
Efficacy	82	10.9%	24	13.3%	106	11.3%
Herd immunity	16	2.1%	0	0.0%	16	1.7%
Strategy	79	10.5%	6	3.3%	85	9.1%
Conspiracy	33	4.4%	36	20.0%	69	7.4%
Freedom of choice	22	2.9%	17	9.4%	39	4.2%
Health Literacy	50	6.6%	0	0.0%	50	5.4%
Other	94	12.5%	4	2.2%	98	10.5%
**Personal Storytelling**	186	24.7%	4	2.2%	190	20.4%
**Tone of voice**						
Neutral	101	13.4%	5	2.8%	106	11.4%
Enthusiastic	59	7.8%	1	0.6%	60	6.4%
Encouraging	54	7.2%	0	0.0%	54	5.8%
Supportive/empathic	32	4.2%	0	0.0%	32	3.4%
Questioning	17	2.2%	0	0.0%	17	1.8%
Polemical/complaining	105	13.9%	142	78.9%	247	26.5%
Worried	27	3.6%	23	12.8%	50	5.3%
Ironic	349	46.3%	9	5.0%	358	38.3%
Other	10	1.3%	0	0.0%	10	1.1%
**Stance**						
Promotional	305	40.5%	0	0.0%	305	32.7%
Neutral	85	11.3%	2	1.1%	87	9.3%
Discouraging	73	9.7%	172	95.6%	245	26.2%
Ambiguous	23	3.1%	0	0.0%	23	2.5%
Indefinite/ironic	256	33.9%	3	1.7%	259	27.7%
Other	12	1.6%	3	1.7%	15	1.6%

The large majority of the videos were about the COVID-19 vaccine (92.6%) and most of them (67%) showed the in-app notice providing access to the WHO or the Italian Ministry of Health’s webpage on COVID-19 vaccines. The in-app notice was triggered by the hashtags used in the video description, therefore it was shown only in one quarter of the Vaccine Sceptics’ videos, as they often did not use vaccine-related hashtags.

### Stance

As for the Top Videos, in most cases the stance was promotional (40.5%). One third of videos had an indefinite-ironic stance and 11.3% were neutral. Only 9.7% of the videos included in this dataset were discouraging, while 3.1% were ambiguous. A high proportion of the promotional videos (43%) were from healthcare professionals.

As expected, almost all of the Vaccine Sceptics’ videos had a discouraging stance (95.6%). Three were indefinite-ironic, two were neutral, and for three the stance was unclear.

Additional file 1 shows users’ and video characteristics by stance, based on a combined dataset (Top Videos + Vaccine Sceptic).

### Tone of voice

For the Top Videos, the most frequent tone of voice was ironic (in almost one half of videos); 13.9% had a polemical tone of voice and 13.4% were neutral. Only 3.6% of videos had a worried tone of voice. For the Vaccine Sceptics’ videos, 78.9% were polemical and 12.8% were worried.

The tone of voice distribution was quite different across stances. Promotional videos were often ironic (28.9%), but also enthusiastic, encouraging, neutral, and polemic were represented. In discouraging videos, tones of voices were mainly polemical (78%) and, less frequently, worried (13.5%). Tone of voice was often neutral in neutral videos (63.2%), but was also classified as ironic in 12.6% of cases. In 93.4% of videos with an indefinite-ironic stance, the tone was ironic.

### Topic

Safety was the most popular topic, both in the Top Videos (50.1%) and in the Vaccine Sceptics’ videos (51.7%).

As for the Top Videos, other popular topics were efficacy (10.9%) and strategy (10.5%). A smaller proportion of videos were on health literacy (6.6%).

As for the Vaccine Sceptics’ videos, the second most popular topic was conspiracy (20%), followed by efficacy (13.3%) and freedom of choice (9.4%).

Considering both datasets, most promotional videos were about safety, followed by efficacy and health literacy. Safety was also the most popular topic among discouraging videos, followed by conspiracy, freedom of choice, and efficacy. In the neutral video category, the most frequent topics were strategy and health literacy. Ambiguous videos were more frequently about strategy and safety.

Almost one quarter of the Top Videos included a reference to personal experiences (24.7%), which were mentioned only in a small portion of the Vaccine Sceptics’ videos (2.2%).

### Source of information

Most of the Top Videos did not report any source of information (81.6%), while almost 10% referred to information from mainstream media.

Among the Vaccine Sceptics’ videos, the source of information was general media in half of the videos and social media in almost 10%. For almost 30% of videos, no information source was reported.

Indefinite-ironic and promotional videos were those which most frequently did not report any source of information (92.7% and 84.6% of the videos respectively), while discouraging and ambiguous videos often mentioned information from general media (44.9% and 43.5% respectively). To a lesser extent, general media information was also mentioned by neutral videos (14.9%). Social media posts were reported as sources in 8.6% of discouraging videos.

The number of videos that reported information from scientific institutions was very low both among Top Videos (3.6%) and among Vaccine Sceptics’ videos (3.3%).

### Video format

Most of the Top Videos were face to camera (67.9%), while 27.6% were just videos or still images with commentary. Conversely, the Vaccine Sceptic users mainly published just videos or images with commentary (63.3%), followed by face to camera (15.6%), infographics (8.9%), and still images (6.6%).

Face to camera videos were the most frequent video format for all stances, with the exception of discouraging videos and videos with a stance classified as “other”, for which videos or images with on-screen text were the most frequent format.

### Stance profiles

**Fig. 1:**
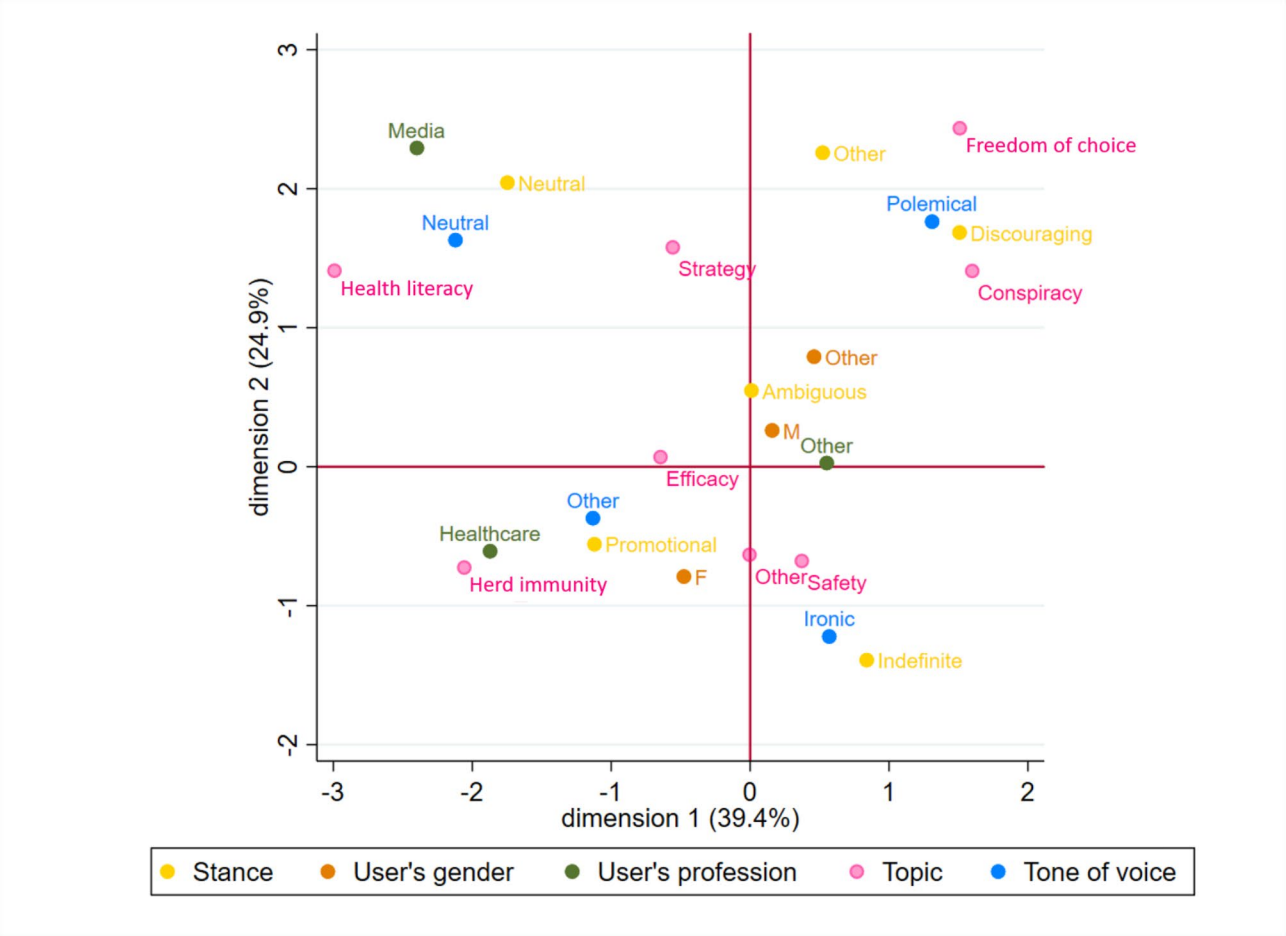
Results of the multiple correspondence analysis

Through correspondence analysis (see Fig. 1), we described typical profiles based on variables associated with the video’s stance. In the figure, each colour represents a variable (e.g. yellow for stance, pink for topic, etc.), and each dot represents a modality for that variable. The smaller the distance between the dots, the higher the association between the variables. This kind of analysis gives a visual representation of variables that are associated with each other, and groups of close variables represent profiles of videos with similar characteristics: if we focus our gaze on the stance dots, other variables’ dots that group around the stance dots represent characteristics that are more frequent in that stance compared to other stances.

Compared to other stances, promotional videos were more frequently about herd immunity and created by healthcare professionals and females. Discouraging videos were more frequently about conspiracy and freedom of choice and had a polemical tone of voice. Neutral videos had a neutral tone of voice and their topic was more frequently health literacy. User’s gender for ambiguous videos was male or classified as other (often couples or families). Indefinite-ironic videos were associated with an ironic tone of voice.

## Discussion

In this study, we report the first investigation on a large dataset of vaccine-related videos shared on TikTok by Italian users. We performed a quantitative and qualitative assessment of the videos that had the highest play count between January 2020 and March 2021. Additionally, we explored the vaccine sceptic community through a manual search of videos with vaccine discouraging content. We ensured a high quality of the classification process by making two distinct researchers, with different professional profiles, classify each video and by discussing all discrepancies collegially.

### Limited vocality of vaccine sceptics on TikTok

While the Italian vaccine sceptics on Twitter have been described as a well connected and self-aware cluster, with users actively mentioning each other [30], we found that the Italian no-vax community on Tiktok is limited in number and vocality. On the other hand, promotional and ironic videos are surely occupying a very large portion of the TikTok vaccine conversation.

Since December 2020, TikTok’s community guidelines “prohibit content that’s false or misleading, including misinformation related to COVID-19, vaccines, and anti-vaccine disinformation” [31]. As a result, content against vaccines is difficult both to publish and to find. Dedicated TikTok teams actively look for false or misleading content or for accounts spreading misinformation [32], and misinformation can also be reported by the platform’s users. Moreover, “searches associated with vaccine or COVID-19 disinformation are redirected to TikTok’s Community Guidelines” and the app “does not autocomplete anti-vaccine hashtags in search”, whereas it does provide direct access to the WHO’s website when users search for COVID-19-related topics. Additionally, TikTok created a COVID-19 information hub hosting authoritative content by the WHO and local health authorities about COVID-19 and vaccines [32]. Given the limited presence of vaccine sceptics in the conversation, TikTok policies against misinformation might actually be more effective compared to those that are adopted by other social media platforms, which is worth further investigation.

### Is the vaccine debate on TikTok less polarised compared to other social media?

Literature has highlighted the different dimensions of (political) polarisation: while ‘ideological’ polarisation refers to the political/ideological distance of ideas and policy platforms, both ‘affective’ [33] and ‘social’ polarisation refer to “a dislike of political opponents and a desire to avoid their company” [34]. While, using the same term ‘polarisation’ might lead one to think that the two dimensions are strongly related, research has shown that social media “may reduce ideological polarisation as a result of leading to higher cross-cutting exposure […] [while] it simultaneously may also be increasing affective polarisation because of the negative nature of these interactions’’ [35].

Some online communities could be described as “polarized but not disconnected” [36], because many social media users “cluster”, but “do not segregate” [36], although research has shown that cross-cutting exposure in the online debate on vaccines is limited. Schmidt et al. [37] reported a strong segregation of the communities involved in the vaccine conversation on Facebook between 2010 and 2017, highlighting a selective exposure of users to polarising content. A strong polarisation has been described also on a large corpus of Twitter messages in English [38] and in a comprehensive analysis of the Italian communities involved in the vaccine discourse on Twitter [30]. Recently, an analysis of content on controversial topics (including vaccination) confirmed the presence of polarised communities both on Facebook and Twitter [39]. Moreover, it has been hypothesised that trolls and bots may amplify the polarisation of the vaccine debate [40].

Our study was not designed to perform a structured analysis of the TikTok communities involved in the vaccine discourse, nor did the kind of data we collected enable us to systematically study the level of cross-cutting exposure between the different groups participating in the debate. Nevertheless, the high amount of videos with an indefinite-ironic stance might indicate that we found an incidence of affective polarisation that is lower than in other social media datasets. Furthermore, based on an unstructured analysis of the comments, we found that conversations were not as divisive as in other social media contexts, and several comments aimed to moderate the tone and highlight the ironic nature of the videos. Indeed, it does not seem that strong ingroup vs. outgroup dynamics are to be found on TikTok with regards to vaccines, as users showing different views are generally not addressed as a strongly disliked group. While these results cannot be generalised, as they only refer to specific timeframe, location and topic, further research is needed to verify if a similar trend might be widespread on TikTok, regarding different topics, cultural contexts and timeframes. We are not assuming that a lower incidence of affective polarisation is a structural feature of a specific platform (TikTok), but we are suggesting that, under the circumstances of our analysis, a lower affective polarisation characterises vaccine-related discussions on TikTok. Future research might focus on understanding how the lower incidence of affective polarisation reflects on the level of segregation of users involved in the vaccine conversation on TikTok, and on the reasons why such a lower affective polarisation characterises vaccine-related conversations on TikTok in comparison with other topics.

### Irony

As previously pointed out, the large presence of videos characterised by an indefinite-ironic stance on TikTok might disrupt the typical mechanisms leading to affective polarisation of the vaccine related conversation seen on other platforms. The indefinite-ironic stance goes beyond the classic dichotomy promotional vs. discouraging, and neither can it be easily categorised as neutral or ambiguous. The need for including this new category in the analysis emerged during the initial coding process, when we identified a large number of videos characterised by a particular brand of irony, sarcasm, parody and humour. Most of these videos represented the user having a fake manic frenzy after being inoculated with the COVID-19 vaccine. At first, we deemed such content as discouraging the vaccination, since it seemed to imply that negative effects were common and to be feared. This initial interpretation was similar to that made by a group of US-based researchers, who screened a lower number of videos (100) in English and Spanish [41], and concluded that the parody-video memes represented “a deliberate and dangerous effort to communicate anti-vaccination sentiment”. Although, research has shown that humour has a weak effect on persuasion [42] - in our case, we believe that the parody videos did not actually increase vaccine scepticism among TikTok users.

During the exploratory phase, we recognized that this kind of videos, rather than discouraging the vaccine, were more likely making fun of the discourses around the vaccine and the COVID-19 health crisis in a complex, layered way. We can hypothesise that these videos somehow ridiculed those who deemed the vaccines unsafe, or contributed to exorcising the fear of vaccines among the platform’s users. Furthermore, in a study on humour in YouTube videos [43], the authors hypothesised that humorous videos might have a role on mitigating tensions and conflicts (and therefore affective polarisation) on the platform.

Moreover, we could describe indefinite-ironic videos as TikTok-specific, as they seem to respond to the peculiar affordances of the platform that encourage “imitation and replication at the platform level” [44], pushing creators to capitalise on current popular trends [45]. Parody videos might also draw a line between accomplished TikTok users (those able to encode and decode the videos and appreciate their irony) and those unfamiliar with TikTok - who will take offence, be dumbfounded, or generally be unable to understand the videos, being unfamiliar with TikTok’s styles and formats, such as lip-syncs and dance routines popular among young people [46]. This is in line with what previously highlighted by Vicari and Murru with regards to the use of irony on Twitter. The authors speculate that, during the first phase of the Italian pandemic, memes and jokes allowed Twitter users to downplay the anxiety triggered by the emergency, “shuffled some of Italy’s traditional divides”, and contributed at the same time to differentiating Twitter users into different publics, as irony “bonds those who get it and alienates those who cannot” [47].

Interestingly, most of the vaccine sceptic creators included in our sample do not seem to be familiar with TikTok’s ironic language. Their tone of voice is often polemical. Also, the format of the Vaccine Sceptics’ videos responds to habits that are not common on TikTok (e.g. less than 2% of discouraging videos are trends or challenges), being more typical of other platforms, like Facebook. This does not mean that vaccine sceptics are not capable of irony or that their content lacks irony, but it suggests that, if such content does exist, it does not find a home on TikTok or in the TikTok’s community of vaccine sceptics.

### Age

The familiarity of TikTok’s users with the platform’s informal usage practices (e.g. use of ironic tones, parodistic performances, catchy music, etc.) is likely affected by sociodemographic factors, including age. It was not possible to assess the exact age of the creators for the videos included in this study, and we cannot exclude that the age distribution of the creators in our dataset differs from that of TikTok users. Nevertheless, more than 50% Italian TikTok users are aged 18–35, and users over 35 are currently a minority on the platform. Young users are more at ease with the platform’s language, thus their content gets more visibility. Overall, the higher popularity of videos with a promotional stance and of those with an indefinite-ironic vaccine stance among the young public of TikTok is in line with Italian COVID-19 vaccination data by age. Throughout the COVID-19 vaccination campaign, the 20–29 age group has always had a higher coverage compared to that reached among individuals 30–59 years [48]. Determinants of the high vaccine acceptance in the youngest age groups certainly deserves further investigation.

### TikTok algorithm

We also believe that the mechanisms of Tiktok’s personalisation algorithms might have a role in shaping the vaccine-related debate on the platform. TikTok’s users receive a very tailored information flow based on videos they may be interested in, according to their preferences, behaviours and other unrevealed criteria. Timeline personalization processes characterise most social media platforms, but TikTok’s algorithms have proved to be more effective in personalising user experiences than their counterparts’ [49]. These algorithms reinforce the so-called “silosociability”, a peculiar sociability, introduced by Tiidenberg et al. to describe Tumblr communities, based on “affect and affinity” and organised around users’ interests and feelings [50]. As a result, Tiktok users may enjoy videos (i.e. indefinite-ironic videos) that are not necessarily shaped by polarised vaccine stances. In other words, users with diverging vaccine stances can enjoy similar videos and share similar environments, as these environments are not characterised by an explicit, polarised approach toward vaccines. This seems to confirm previous findings suggesting that conversations around vaccines on social media platforms are moving “beyond polarisation” [51].

### Safety

Another interesting result is the high prevalence of videos focusing on safety issues, both in the Top Videos and in the Vaccine Sceptics’ videos.

The popularity of the safety topic in the Top Videos is partly due to the high proportion of videos reporting a parody of vaccine side effects, discussed in the previous section. Other recurring themes related to safety among the Top Videos include: explanation of how the vaccine works; updates on vaccine trials; storytimes of the user’s vaccine experience, with a description of the reported symptoms after the vaccination. Most of these videos have a supportive and quiet tone, and aim at reassuring other users on the vaccines’ composition and on their side effects. Commonly, the creators do not take a defensive tone, and tend to respond to sceptical comments with irony, scientific data, and a slightly argumentative tone. We did not find any video openly criticising vaccine sceptics.

Among the discouraging videos in the top video dataset, the main concerns regarded, in general, the “experimental” nature of the vaccine (including its composition), and, more specifically, potential side effects, commonly claimed to be induced by a genetic mutation triggered by the vaccine, which included sterility, autism, and even homosexuality. The suspension of the Astrazeneca/Vaxzevria vaccine in Europe raised concerns for several users. The most popular video in this sample is about a nurse who fainted after receiving the COVID-19 shot. Moreover, the fact that some healthcare professional refused to take the vaccine was seen as supporting the claim of its lack of safety.

When exploring the videos from the sceptic community, the tones of the conversations were mainly polemical, defensive, sometimes defiant. Here, the side effects claimed to be associated with the vaccine were variable and included death, sterility, and seizures. Many creators included videos of people having a fit after the vaccine, with the aim of inducing fear in those who watched. Most of the safety concerns had a conspiracy nuance: commonly the creators blamed institutions (including medicine agencies) and healthcare professionals, hypothesising the existence of safety data that were hidden by pharmaceutical companies and the government. Also those who decided to take the vaccine were blamed for being “enslaved to strong powers”, and were ironically pictured as a herd of sheep. Few discouraging videos with a high play count were trends or challenges, while no video from the no-vax community was characterised by this peculiar Tiktok style (which is clearly favoured by the algorithm).

Interestingly, 90% of the videos with a discouraging stance, for which the creator’s gender was identifiable, were created by males. This is confirmed by the results of the multiple correspondence analysis, which showed a higher proportion of females among creators with a promotional stance compared to other stances. Before the pandemic, a survey showed that females were more favourable to vaccines than males in Italy [52]. On the other hand, with regard to the COVID-19 vaccine, female gender was associated with vaccine refusal based on a more recent Italian survey conducted among the elderly [53]. According to a large survey conducted in December 2020 in several countries with a high COVID-19 burden [54], no gender differences were identified in vaccine acceptance in Italy. The differences in these results could be explained by the timing of the surveys as well as by the sociodemographic characteristics of Tiktok users. A wider investigation conducted on a more representative sample of the Italian population and on users of different social media platforms could better highlight the role of gender and other determinants on vaccine acceptance.

### Non experts and healthcare professionals

One of the concerns related to the infodemic is the presence of several non-expert figures providing vaccine related-information on social media. This phenomenon is based on the so-called ‘easiness effect’, for which people tend to ‘underestimate their dependence on experts and conclude that they are capable of evaluating the veracity, relevance, and sufficiency of the contents’ [55]. In a previous study on the Italian vaccine conversation on Twitter, before and during the first phase of the pandemic, a very limited presence of healthcare professionals (1.5%) has been reported [56]. The higher representation of Italian healthcare professionals on TikTok (13.4%) could be biassed by the different sampling methods used in these two studies: in the Twitter study, a random sample of approximately 3000 tweets was analysed, while the sample used in the present study included videos with a high play count, which might have selected videos created by healthcare professionals. Boatman et al. showed that 30% of a sample of 170 top HPV vaccine-related TikTok videos in English were by healthcare professionals [57]. Healthcare professionals seem to be often capable of understanding and exploiting Tiktok’s characteristics, posting videos that include humour, self-criticism, and specific health content in meme-like forms, which could effectively target a young audience [58]. An interesting initiative on communication by healthcare professionals on social media is #TeamHalo [59], a network of scientists and healthcare professionals from around the world, aiming to address concerns and misinformation on the COVID-19 pandemic on social media on a voluntary basis. In Italy, healthcare professionals also convey vaccine related information through more traditional, web-based channels, such as vaccinarsi.org [29] and dottoremaeveroche.it [29, 60], both funded by public health entities.

### Limitations

Our study has a number of limitations.

Our work is based on the analysis of two datasets, obtained through two different techniques: the first dataset included the videos that performed better on the platform in terms of sharing, while the second one was obtained by a manual exploration of the vaccine-sceptic community. This aspect of our methodology implies that the generalisability of our results to the whole population of TikTok users is limited, and we cannot assume that the users we identified through our search are representative of those involved in the Italian vaccine-related conversation on TikTok. Nevertheless, considering the videos gathered through the API search only, while being an apparently more sound sampling method, would have further under-represented Vaccine Sceptics’ videos, who often avoid the use of vaccine-related hashtags. On the other hand, one strength of our study is the size of the sample of videos included in our dataset, which, to our knowledge, is larger than that of any previous study on TikTok, thus improving the precision of our estimates and the reliability of our results.

Despite the generalisability issue, we believe that understanding the characteristics of videos with the highest performance and popularity can be useful to inform communication initiatives that may take into account the use of this platform. Moreover, monitoring the sceptic community as we did in the present study can help public health institutions to track the circulation of conspiracy theories and other forms of misinformation and disinformation.

Other methodological limitations should be considered. Research on TikTok is still limited, and no established literature exists on the quantitative and qualitative analysis on this social platform. Therefore, we had no validated methodology to follow and some of our results might be biassed.

## Conclusions

In conclusion, our analysis suggests a particular character of the Italian vaccine conversation on TikTok compared to other social media platforms. First, we found that the vaccine sceptic community has limited presence and vocality on TikTok in Italy. In our sample, vaccine sceptics often use a polemical tone of voice which is not common on the platform (nor is it favoured by the algorithm), and tend to segregate themselves from the public conversation, probably to avoid banning due to TikTok’s policies against misinformation. To this aim, they often avoid the use of specific vaccine-related hashtags. Snowball sampling - or alternative methods - are therefore needed to study the vaccine sceptic community on this platform. In the Italian context, the typical polarisation of the vaccine debate seen on other social media might be disrupted on TikTok, mainly by the large presence of ironic videos making fun of common fears around vaccines. These kinds of videos, which are characterised by a specific TikTok style (trends, catchy music), seem to be favoured by the algorithm, and might have a mitigating effect on diverging opinions. Safety topics were popular on the platform, among promotional, discouraging, and ironic videos. We recorded an interesting presence of creators working in healthcare, who seem to be at ease with the platform’s popular trends and characteristics.

These observations suggest that TikTok could be an interesting medium to be considered with regards to vaccine communication, both as a source of information on the communities involved in the conversation and on popular trends and topics, as well as a means to deliver vaccine promotion campaigns, especially those targeting adolescents and young adults. The use of irony in the vaccine conversation and its effect on message informativeness and vaccine acceptance across different cultures deserves further research.

## Electronic supplementary material

Below is the link to the electronic supplementary material.

Supplementary Material 1

## Data Availability

The datasets used and/or analysed during the current study are available from the corresponding author on reasonable request.
