# Supplementary material for: Exploring the vaccine conversation on TikTok in Italy: beyond classic vaccine stances
Source: BMC Public Health. 2023 May 12;23:880. doi: 10.1186/s12889-023-15748-y (PMC10176305; doi:10.1186/s12889-023-15748-y)
Supplement: Supplementary file 1 — Supplementary Material 1 [file 12889_2023_15748_MOESM1_ESM.docx]

**Users’ and video characteristics by stance (combined dataset)**

|  | **Promotional** | | **Neutral** | | **Discouraging** | | **Ambiguous** | | **Indefinite/**  **ironic** | | **Altro** | |
| --- | --- | --- | --- | --- | --- | --- | --- | --- | --- | --- | --- | --- |
|  | n | % | n | % | n | % | n | % | n | % | n | % |
| **Sex** |  |  |  |  |  |  |  |  |  |  |  |  |
| Male | 163 | 54.3% | 49 | 57.6% | 159 | 79.5% | 11 | 50.0% | 159 | 63.6% | 10 | 71.4% |
| Female | 128 | 42.7% | 21 | 24.7% | 15 | 7.5% | 10 | 45.5% | 69 | 27.6% | 4 | 28.6% |
| Other  (profiles from organisations, families etc.) | 9 | 3.0% | 15 | 17.6% | 26 | 13.0% | 1 | 4.5% | 22 | 8.8% | 0 | 0.0% |
| **User's profession** |  |  |  |  |  |  |  |  |  |  |  |  |
| Health care professional | 131 | 43.0% | 15 | 17.2% | 2 | 0.9% | 0 | 0.0% | 8 | 3.1% | 1 | 6.7% |
| Media and journalism | 10 | 3.3% | 16 | 18.4% | 0 | 0.0% | 1 | 4.3% | 5 | 1.9% | 1 | 6.7% |
| Other | 164 | 53.8% | 56 | 64.4% | 220 | 99.1% | 22 | 95.7% | 246 | 95.0% | 13 | 86.7% |
| **User's followers, median (IQR)** | 91324.3  (5426 - 124650) | | 69499.5  (4827-92800) | | 6683.6  (336-6527) | | 23900  (8935-33400) | | 90992.7  (3096-72750) | | 17002.5  (8854-23125.5) | |
| **Play count** | 27600  (13600-62300) | | 23200  (13100-41200) | | 2673  (145-15200) | | 34500  (19400-46300) | | 23300  (12000-60700) | | 15200  (12800-22900) | |
| **Comment count** | 107 (41 - 259) | | 57 (29 - 176) | | 8 (0 - 78) | | 73 (27 - 144) | | 44 (15 - 115) | | 100 (40 - 261) | |
| **Type of vaccine** |  |  |  |  |  |  |  |  |  |  |  |  |
| anti-COVID-19 | 272 | 89.2% | 79 | 90.8% | 228 | 93.1% | 21 | 91.3% | 252 | 97.3% | 13 | 86.7% |
| other | 33 | 10.8% | 8 | 9.2% | 17 | 6.9% | 2 | 8.7% | 7 | 2.7% | 2 | 13.3% |
| **COVID-19 or COVID-19 vaccine banner** | 247 | 81.0% | 70 | 80.5% | 98 | 40.0% | 17 | 73.9% | 186 | 71.8% | 8 | 53.3% |
| **Q&A style** | 69 | 22.6% | 14 | 16.1% | 21 | 8.6% | 4 | 17.4% | 16 | 6.2% | 3 | 20.0% |
| **Trend or challenge style** | 40 | 13.1% | 7 | 8.0% | 4 | 1.6% | 2 | 8.7% | 103 | 39.8% | 1 | 6.7% |
| **Engaging music/lip sync** | 166 | 54.4% | 37 | 42.5% | 102 | 41.6% | 12 | 52.2% | 151 | 58.3% | 8 | 53.3% |
| **Video format** |  |  |  |  |  |  |  |  |  |  |  |  |
| Face to Camera | 238 | 78.0% | 46 | 52.9% | 54 | 22.0% | 13 | 56.5% | 184 | 71.0% | 5 | 33.3% |
| Video or image with on-screen text | 63 | 20.7% | 31 | 35.6% | 148 | 60.4% | 8 | 34.8% | 62 | 23.9% | 10 | 66.7% |
| Video without text | 4 | 1.3% | 3 | 3.4% | 14 | 5.7% | 0 | 0.0% | 11 | 4.2% | 0 | 0.0% |
| Image without text | 0 | 0.0% | 3 | 3.4% | 13 | 5.3% | 1 | 4.3% | 0 | 0.0% | 0 | 0.0% |
| Infographic | 0 | 0.0% | 4 | 4.6% | 16 | 6.5% | 1 | 4.3% | 2 | 0.8% | 0 | 0.0% |
| **Information source** |  |  |  |  |  |  |  |  |  |  |  |  |
| Social media posts | 3 | 1.0% | 0 | 0.0% | 21 | 8.6% | 1 | 4.3% | 5 | 1.9% | 0 | 0.0% |
| Media | 15 | 4.9% | 13 | 14.9% | 110 | 44.9% | 10 | 43.5% | 9 | 3.5% | 6 | 40.0% |
| Non scientific institutions | 4 | 1.3% | 3 | 3.4% | 2 | 0.8% | 0 | 0.0% | 2 | 0.8% | 1 | 6.7% |
| Scientific institutions | 11 | 3.6% | 6 | 6.9% | 3 | 1.2% | 0 | 0.0% | 0 | 0.0% | 0 | 0.0% |
| Unspecified source of information | 14 | 4.6% | 6 | 6.9% | 16 | 6.5% | 1 | 4.3% | 3 | 1.2% | 0 | 0.0% |
| No information source reported | 258 | 84.6% | 59 | 67.8% | 92 | 37.6% | 11 | 47.8% | 240 | 92.7% | 8 | 53.3% |
| **Video's topic** |  |  |  |  |  |  |  |  |  |  |  |  |
| Safety | 142 | 46.6% | 12 | 13.8% | 126 | 51.4% | 6 | 26.1% | 185 | 71.4% | 0 | 0.0% |
| Efficacy | 58 | 19.0% | 6 | 6.9% | 29 | 11.8% | 2 | 8.7% | 10 | 3.9% | 1 | 6.7% |
| Herd immunity | 15 | 4.9% | 1 | 1.1% | 0 | 0.0% | 0 | 0.0% | 0 | 0.0% | 0 | 0.0% |
| Strategy | 21 | 6.9% | 27 | 31.0% | 10 | 4.1% | 10 | 43.5% | 9 | 3.5% | 8 | 53.3% |
| Conspiracy | 8 | 2.6% | 2 | 2.3% | 48 | 19.6% | 1 | 4.3% | 9 | 3.5% | 1 | 6.7% |
| Freedom of choice | 2 | 0.7% | 5 | 5.7% | 27 | 11.0% | 2 | 8.7% | 2 | 0.8% | 1 | 6.7% |
| Health Literacy | 31 | 10.2% | 19 | 21.8% | 0 | 0.0% | 0 | 0.0% | 0 | 0.0% | 0 | 0.0% |
| Other | 28 | 9.2% | 15 | 17.2% | 5 | 2.0% | 2 | 8.7% | 44 | 17.0% | 4 | 26.7% |
| **Personal Storytelling** | 114 | 37.5% | 9 | 10.3% | 8 | 3.3% | 3 | 13.0% | 54 | 20.8% | 2 | 13.3% |
| **Tone of voice** |  |  |  |  |  |  |  |  |  |  |  |  |
| Neutral | 33 | 10.8% | 55 | 63.2% | 7 | 2.9% | 2 | 8.7% | 7 | 2.7% | 2 | 13.3% |
| Enthusiastic | 57 | 18.7% | 0 | 0.0% | 1 | 0.4% | 1 | 4.3% | 1 | 0.4% | 0 | 0.0% |
| Encouraging | 51 | 16.7% | 1 | 1.1% | 0 | 0.0% | 0 | 0.0% | 2 | 0.8% | 0 | 0.0% |
| Supportive/empathic | 28 | 9.2% | 4 | 4.6% | 0 | 0.0% | 0 | 0.0% | 0 | 0.0% | 0 | 0.0% |
| Questioning | 8 | 2.6% | 2 | 2.3% | 2 | 0.8% | 4 | 17.4% | 1 | 0.4% | 0 | 0.0% |
| Polemical/complaining | 33 | 10.8% | 8 | 9.2% | 191 | 78.0% | 4 | 17.4% | 1 | 0.4% | 10 | 66.7% |
| Worried | 6 | 2.0% | 4 | 4.6% | 33 | 13.5% | 5 | 21.7% | 1 | 0.4% | 1 | 6.7% |
| Ironic | 88 | 28.9% | 11 | 12.6% | 9 | 3.7% | 6 | 26.1% | 242 | 93.4% | 2 | 13.3% |
| Other | 1 | 0.3% | 2 | 2.3% | 2 | 0.8% | 1 | 4.3% | 4 | 1.5% | 0 | 0.0% |
